# Comparative evaluation of definitive therapy for deep-seated *Stenotrophomonas maltophilia* infections

**DOI:** 10.1093/jacamr/dlaf215

**Published:** 2025-11-19

**Authors:** Hunter Curry, Danielle Casaus, Kristen Lucas, Ashlan J Kunz Coyne

**Affiliations:** Department of Pharmacy Services, University of Kentucky HealthCare, Lexington, KY, USA; Department of Pharmacy Services, University of Kentucky HealthCare, Lexington, KY, USA; Department of Pharmacy Practice and Science, University of Kentucky College of Pharmacy, Lexington, KY, USA; Department of Pharmacy Practice and Science, University of Kentucky College of Pharmacy, Lexington, KY, USA

## Abstract

**Background:**

*Stenotrophomonas maltophilia*, a MDR pathogen, causes healthcare-associated infections in critically ill patients. Deep-seated infections pose treatment challenges due to high bacterial inocula and biofilms. Prior studies often include polymicrobial or colonization cases, complicating evaluations. This study compares monotherapy versus combination therapy in deep-seated, monomicrobial infections to clarify optimal treatment.

**Methods:**

This single centre, retrospective study evaluated patients admitted from 2010 to 2023 with deep-seated, monomicrobial *S. maltophilia* infections receiving *in vitro* active antimicrobials within 72 h of culture collection. The primary outcome was clinical failure, defined as 30-day all-cause mortality, infection-related readmission, or recurrent infection. Regression models used inverse probability of treatment weighting and time-varying covariates.

**Results:**

Among 190 patients, 99 (52.1%) received monotherapy and 91 (47.9%) received combination therapy. Clinical failure occurred in 30.3% of monotherapy and 34.1% of combination therapy patients (*P* = 0.579). In propensity-weighted regression, combination therapy with levofloxacin plus minocycline (adjusted odds ratio [aOR] 0.44, 95% CI 0.17–0.94, *P* = 0.022) and levofloxacin plus trimethoprim/sulfamethoxazole (aOR 0.19, 95% CI 0.08–0.76, *P* = 0.035) was associated with reduced odds of clinical failure. Higher SOFA scores (aOR 1.89, 95% CI 1.25–2.12, *P* = 0.033), prior carbapenem use (aOR 2.02, 95% CI 1.54–2.24, *P* = 0.014) and empyema (aOR 2.77, 95% CI 2.18–3.96, *P* = 0.011) increased the odds of clinical failure.

**Conclusion:**

Levofloxacin-based combination therapies may reduce clinical failure in deep-seated *S. maltophilia* infections, while SOFA scores, carbapenem use and empyema increase risk. Prospective trials are warranted to confirm the efficacy of levofloxacin-based combinations.

## Introduction

*Stenotrophomonas maltophilia* is an opportunistic, MDR, Gram-negative pathogen increasingly implicated in healthcare-associated infections among critically ill and immunocompromised patients.^1–5^ Its intrinsic resistance, mediated by β-lactamase production, efflux pumps and other MDR mechanisms, severely limits therapeutic options, complicating clinical management.^2,4–6^ Despite being historically considered a low-virulence organism, *S. maltophilia* is associated with high mortality rates (21%–69%), particularly in patients with pneumonia, septic shock, or ICU admission.^6–8^ Distinguishing true infection from colonization remains a critical challenge, especially in polymicrobial respiratory and wound isolates, which can lead to inappropriate use of antimicrobials.^2,4,6,9,10^

While pneumonia and bacteremia are the most reported *S. maltophilia* infections, deep-seated infections such as bone and joint infections, deep abscesses, empyema and infective endocarditis pose unique treatment challenges.^2,3,5^ These infections often involve high bacterial inocula and biofilm formation, increasing the risk of treatment failure and recurrence.^11^ Limited pharmacokinetic/pharmacodynamic (PK/PD) data and the need for prolonged therapy further complicate management, often necessitating combination regimens to achieve bacteria eradication.^4,11–13^

Current treatment strategies rely on a limited set of antimicrobials with *in vitro* activity against *S. maltophilia*, primarily trimethoprim/sulfamethoxazole, levofloxacin and minocycline.^6,12,14^ Trimethoprim/sulfamethoxazole was historically regarded as the first-line agent, though more recent data support levofloxacin and minocycline as appropriate first-line alternatives.^15^ The Infectious Diseases Society of America (IDSA) recommends empiric combination therapy for moderate-to-severe *S. maltophilia* infections until susceptibility information is available, based on limited observational studies.^14^ Existing studies often fail to differentiate monomicrobial from polymicrobial infections, include potential cases of colonization, or lack standardized definitions of definitive therapy, creating uncertainty about optimal treatment of *S. maltophilia* infections.^16–21^

This study addresses these gaps by comparing clinical outcomes of monotherapy versus combination therapy for deep-seated, monomicrobial *S. maltophilia* infections. Focusing on deep-seated, monomicrobial infections will reduce the risk of colonization misclassification and confounding from co-pathogens, which may clarify the direct impact of *S. maltophilia*-directed therapy. With well-defined infections and rigorous treatment criteria, we aim to provide evidence-based insights to guide therapeutic decisions and improve outcomes in this challenging patient population.

## Methods

This retrospective study analysed adult patients (≥18 years) admitted to University of Kentucky HealthCare academic medical centres between September 2010 and December 2023 with deep-seated, monomicrobial *S. maltophilia* infections. Eligible patients had *S. maltophilia* isolated from a deep-seated culture and were required to receive an *in vitro* active antimicrobial within 72 h of culture collection.

For patients where the clinical team could not obtain a culture from the expected site of infection due to the invasiveness of the procedure or patient instability, a positive blood culture with clinical correlation to a deep-seated source was required. Clinical correlation was determined by imaging (e.g. X-ray, CT, MRI, or ultrasound) confirming a deep-seated infection, relevant clinical symptoms (e.g. fever, localized pain, or organ dysfunction) and clinician documentation in the medical record linking the bacteremia to a deep-seated source.

Deep-seated infections included bone and joint infections, deep abscesses (intra-abdominal, hepatic, pancreatic, psoas, lung, mediastinal, sub-fascial, or perinephric), empyema and infective endocarditis (including cases with septic emboli). Infections were identified using ICD-9/10 codes and confirmed by specimens obtained via surgery, incision and drainage, or imaging-guided puncture (ultrasound or CT) from normally sterile sites. Bone infections required confirmation by imaging (X-ray, MRI or CT) showing osteomyelitis or joint involvement and/or microbiological evidence from surgically obtained bone or tissue cultures. Immunocompromising conditions were defined as chemotherapy or radiation therapy within the prior 30 days, HIV/AIDS with CD4 count <200 cells/mm³, or corticosteroid use (≥14 continuous days at >40 mg/day prednisone equivalent).

All microbiological identifications and susceptibility testing followed standard procedures with susceptibility interpreted per CLSI guidelines.^22^ For antimicrobials with evolving breakpoints during the study period (2010–23), *in vitro* susceptibility was determined using the CLSI criteria in effect at the time of treatment initiation to account for changes. Agents included trimethoprim/sulfamethoxazole (8–15 mg/kg/day of the trimethoprim component every 8–12 h), levofloxacin (750 mg every 24 h) and minocycline (200 mg every 12 h).^14,22^ Renal function adjustments were applied as needed. Combination therapy was defined as two susceptible agents for ≥70% of treatment duration; definitive therapy was the regimen with *in vitro* activity used for ≥70% of the course if therapies transitioned.

Source control, including surgical or percutaneous drainage, debridement, or valve replacement, was performed in all patients to eliminate infection foci. Success was confirmed within 14 days post-intervention, or at therapy completion if earlier assessments were inconclusive, using one or more of the following criteria: (i) clinical improvement (e.g. resolution of fever or symptoms), (ii) microbiological clearance (e.g. negative follow-up cultures), (iii) radiological improvement (e.g. reduction in abscess size) or (iv) clinician consensus documented in the medical record indicating resolution or significant improvement.

Exclusion criteria included death or transfer to hospice within 48 h of admission, transfer from an outside hospital with a positive *S. maltophilia* culture, polymicrobial cultures, concomitant infections with organisms other than *S. maltophilia*, non-loculated respiratory tract infections and non-sterile wound cultures. Respiratory cultures associated with ICD-9/10 diagnoses of empyema or lung abscess and wound cultures from sterile debridement were included.

The primary outcome was clinical failure, a composite of 30-day all-cause mortality (death from any cause within 30 days of initiating active antimicrobials), 30-day infection-related readmission (hospital readmission within 30 days of discharge for *S. maltophilia* infection treatment), or recurrent infection (positive *S. maltophilia* culture from blood or the index site within 30 days post-definitive therapy). For recurrent infection, initial clearance of the infection could be determined by the clinical team’s documentation in the medical record stating the infection was considered resolved, without requiring a repeat culture. Secondary outcomes included individual components of the composite outcome and 90-day infection-related readmission.

Clinical failure estimates (40% for monotherapy, 25% for combination therapy) were based on prior studies of S. maltophilia infections, including reports of higher failure rates with monotherapy compared to combination therapy.^17,23^ The sample size (*N* = 190) provided 80% power at 95% confidence to detect a 15% difference in clinical failure rates, with all eligible patients from 2010 to 2023 included to maximize statistical power. Continuous variables, reported as medians with IQR, were analysed using Mann–Whitney *U*-test (non-parametric) or *t*-tests (parametric); categorical variables, as *n* (%), used Pearson’s χ^2^ or Fisher’s exact tests. Descriptive analyses evaluated primary and secondary outcomes. Variables with *P* < 0.2 in univariable analyses entered multivariable logistic regression to identify clinical failure predictors. Inverse probability of treatment weighting (IPTW) with propensity scores (covariates: age, sex, BMI, hospital-acquired infection, immunocompromised status, SOFA score; time-varying: year of definitive therapy initiation, days from active therapy initiation to source control) was used to control for confounding. Selection of covariates for IPTW was informed by clinical relevance and prior literature. Collinearity among candidate variables was assessed using variance inflation factors, with a cut-off of <2 required for inclusion. Hospital-acquired infection was defined as the collection of the positive index culture after 48 h of hospital admission. Covariate balance was assessed using standardized mean differences (SMD <0.1). Stabilized IPTW weights balanced groups. Models reported OR and adjusted OR (aOR) with 95% CI (*P* < 0.05). Analyses used SPSS (version 28.0, Armonk, NY) and R (version 4.3.0, Vienna, Austria). The University of Kentucky Institutional Review Board approved the study. Data were extracted from electronic medical records with manual validation.

## Results

Of the 190 patients meeting inclusion criteria for review, 99 (52.1%) received monotherapy and 91 (47.9%) received combination therapy. Table 1 presents baseline demographics and clinical characteristics. The median age was 54 years (IQR 42–64), with 62.6% male representation. More than half of patients had hospital-acquired *S. maltophilia* infections (52.1%), were treated in an ICU setting (52.1%), or had concomitant *S. maltophilia* bacteremia (57.4%). Hospital-acquired infections were significantly more frequent in the monotherapy group (59.6% versus 44.0%, *P* = 0.031). This cohort was critically ill, with more than half of patients requiring ICU-level care and had a high baseline risk of mortality. Acute severity was reflected by a median APACHE II score of 16 (IQR 12–20) and SOFA score of 3 (IQR 2–4.3), while baseline risk was reflected by a Charlson Comorbidity Index of 4 (IQR 3–5). Immunocompromised patients comprised 22.6% of the cohort. Infectious Diseases (ID) consultation occurred in 45.3% of all cases. The most common infection sites were bone and joint (33.7%), deep abscesses (33.2%), empyema (28.4%) and infective endocarditis (4.7%; 1/9 culture-positive, with the remainder diagnosed by Modified Duke Criteria [e.g. clinical findings, bacteremia and imaging]). Monotherapy primarily consisted of levofloxacin (43/99, 22.6%) or trimethoprim/sulfamethoxazole (41/99, 21.6%), while combination regimens were most commonly levofloxacin plus minocycline (31/91, 16.3%), levofloxacin plus trimethoprim/sulfamethoxazole (30/91, 15.8%), or trimethoprim/sulfamethoxazole plus minocycline (30/91, 15.8%). The median duration of definitive therapy was similar between groups (25 days, IQR 19–30.3).

**Table 1.** Baseline admission and treatment characteristics

	*N* = 190	Monotherapy (*n* = 99)	Combination (*n* = 91)	*P* value
Age, years	54 (42–64)	53 (40–64)	54 (42–62)	0.838
Male sex	119 (62.6%)	64 (64.6%)	55 (60.4%)	0.549
BMI (kg/m^2^)	27.1 (22.9–33.5)	27.1 (23.1–32.9)	27.1 (22.7–33.8)	0.945
Weight (kg)	79.5 (64.7–99.2)	78.9 (63.5–104.3)	79.9 (65.7–98.6)	0.943
Height (m)	1.7 (1.6–1.8)	1.7 (1.6–1.8)	1.7 (1.6–1.8)	0.578
Race				0.627
White	171 (90%)	88 (88.9%)	83 (91.2%)	
Black or African American	15 (7.9%)	9 (9.1%)	6 (6.6%)	
Spanish American	3 (1.6%)	2 (2%)	1 (1.1%)	
American Indian or Alaskan	1 (0.5%)	0 (0%)	1 (1.1%)	
Admission last 90 days	73 (38.4%)	37 (37.4%)	36 (39.6%)	0.757
Hospital-acquired infection	99 (52.1%)	59 (59.6%)	40 (44%)	0.031
Admit to culture collection (days)	2.3 (0.4–9.2)	4.6 (0.7–12.2)	1.11 (0.2–6.8)	0.006
ID consulted	86 (45.3%)	47 (47.5%)	39 (42.9%)	0.523
ICU admission	99 (52.1%)	49 (49.5%)	50 (54.9%)	0.453
Immunocompromised	43 (22.6%)	24 (24.2%)	19 (20.9%)	0.580
SOFA score	3 (2–4.3)	3 (2–4)	4 (2–5)	0.101
APACHE-II score	16 (12–20)	15 (11–19)	16 (13–20)	0.239
Charlson comorbidity index	4 (3–5)	4.0 (3–5)	4 (3–5)	0.909
Length of hospital stay (days)	13.4 (8–25.1)	15.5 (8–31.3)	11.3 (8–22.5)	0.301
Duration of therapy (days)^a^	25 (19–30.3)	25 (20–31)	23 (18–30)	0.213
Antimicrobial use last 90 days				
Fluoroquinolone	34 (17.9%)	19 (19.2%)	15 (16.5%)	0.627
Aminoglycoside	24 (12.6%)	13 (13.1%)	11 (12.1%)	0.630
Carbapenem	28 (14.7%)	18 (18.2%)	10 (11%)	0.162
Source				
Bone and joint	64 (33.7%)	35 (35.4%)	29 (31.9%)	0.612
Deep abscess	63 (33.2%)	36 (36.4%)	27 (29.7%)	0.328
Empyema	54 (28.4%)	23 (23.2%)	31 (34.1%)	0.098
Infective endocarditis^b^	9 (4.7%)	5 (5.1%)	4 (4.4%)	0.553
Concomitant bacteremia	109 (57.4%)	58 (58.6%)	51 (56.0%)	0.723
Definitive antimicrobial therapy^c^				
LVX	43 (22.6%)	43 (43.4%)		
SXT	41 (21.6%)	41 (41.4%)		
MIN	15 (7.9%)	15 (15.2%)		
LVX + MIN	31 (16.3%)		31 (34.1%)	
LVX + SXT	30 (15.8%)		30 (33%)	
SXT + MIN	30 (15.8%)		30 (33%)	

Data represented as median (IQR) for continuous variables and *n* (%) for categorical variables.

ID, infectious diseases; APACHE, acute physiology and chronic health evaluation; LVX, levofloxacin; SXT, trimethoprim/sulfamethoxazole; MIN, minocycline.

^a^Duration of therapy (days) reflects the total duration of all active antimicrobial therapy received during the treatment course, not only the definitive regimen.

^b^Of the nine patients with infective endocarditis, one had culture-positive *S. maltophilia* endocarditis; eight were diagnosed based on Modified Duke Criteria (e.g. clinical findings, bacteremia and imaging).

^c^All patients received an *in vitro* active antimicrobial within 72 h of culture collection.

Primary and secondary outcomes are reported in Tables 2 and 3. Clinical failure, the primary composite outcome of 30-day mortality, infection-related readmission, or recurrent infection, occurred in 30/99 monotherapy patients (30.3%) and 31/91 combination therapy patients (34.1%; *P* = 0.579). Within this composite, though none were statistically significant, 30-day mortality was higher for combination therapy (18/91, 19.8%) than monotherapy (10/99, 10.1%; *P* = 0.060), while 30-day recurrent infection (16/99, 16.2% versus 9/91, 9.9%; *P* = 0.201) and readmission (21/99, 21.2% versus 13/91, 14.3%; *P* = 0.213) were higher for monotherapy.

**Table 2.** Clinical outcomes

	*N* = 190	Monotherapy (*n* = 99)	Combination (*n* = 91)	*P* value
Clinical failure^a^	61 (32.1)	30 (30.3)	31 (34.1)	0.579
30-day mortality	28 (14.7)	10 (10.1)	18 (19.8)	0.060
30-day infection-related readmission	34 (17.9)	21 (21.2)	13 (14.3)	0.213
30-day recurrent infection	25 (13.2)	16 (16.2)	9 (9.9)	0.201
Recurrent infection with resistance	5 (2.6)	3 (3)	2 (2.2)	0.721
90-day infection-related readmission	50 (26.3)	30 (30.3)	20 (22.0)	0.193

Data represented as *n* (%).

^a^Clinical failure: 30-day all-cause mortality (death within 30 days of initiating active antimicrobials), 30-day infection-related readmission (hospital readmission within 30 days of discharge for *S. maltophilia* infection treatment), or recurrent infection (positive *S. maltophilia* culture from blood or the index site within 30days post-definitive therapy).

**Table 3.** Clinical outcomes by treatment group

	Monotherapy (*n* = 99)			Combination (*n* = 91)			*P* value
	LVX (*n* = 43)	SXT (*n* = 41)	MIN (*n* = 15)	LVX + MIN (*n* = 31)	LVX + SXT (*n* = 30)	SXT + MIN (*n* = 30)	
Clinical failure^a^	14 (32.6%)	12 (29.3%)	4 (26.7%)	6 (19.4%)	16 (53.3%)	9 (39.0%)	0.579
30-day mortality	4 (9.3%)	4 (9.8%)	2 (13.3%)	5 (16.1%)	10 (33.3%)	3 (10.0%)	0.060
30-day infection-related readmission	11 (25.6%)	8 (19.5%)	2 (13.3%)	1 (3.2%)	6 (20.0%)	6 (20.0%)	0.213
30-day recurrent infection	9 (20.9%)	5 (12.2%)	2 (13.3%)	1 (3.2%)	4 (13.3%)	4 (13.3%)	0.201
90-day infection-related readmission	14 (32.6%)	12 (29.3%)	4 (26.7%)	6 (19.4%)	5 (16.7%)	9 (30.0%)	0.193

Data represented as *n* (%).

LVX, levofloxacin; SXT, trimethoprim/sulfamethoxazole; MIN, minocycline.

^a^Clinical failure: 30-day all-cause mortality (death within 30 days of initiating active antimicrobials), 30-day infection-related readmission (hospital readmission within 30 days of discharge for *S. maltophilia* infection treatment), or recurrent infection (positive *S. maltophilia* culture from blood or the index site within 30 days post-definitive therapy).

In the propensity-weighted binary regression model (Table 4), combination therapy with levofloxacin plus minocycline (aOR 0.44, 95% CI 0.17–0.94, *P* = 0.022) and levofloxacin plus trimethoprim/sulfamethoxazole (aOR 0.19, 95% CI 0.08–0.76, *P* = 0.035) was associated with reduced odds of clinical failure. Conversely, higher SOFA scores (aOR 1.89, 95% CI 1.25–2.12, *P* = 0.033), carbapenem use in the preceding 90 days (aOR 2.02, 95% CI 1.54–2.24, *P* = 0.014) and presence of empyema (aOR 2.77, 95% CI 2.18–3.96, *P* = 0.011) were associated with increased odds of clinical failure.

**Table 4.** Binary regression for clinical failure

	Unadjusted cohort		Propensity score IPTW cohort^a^	
	OR (95% CI)	*P* value	aOR (95% CI)	*P* value
LVX (reference group)		0.449		0.151
SXT	0.76 (0.24–2.45)	0.648	1.62 (0.71–2.99)	0.169
MIN	0.81 (0.25–2.67)	0.735	0.87 (0.44–1.72)	0.691
LVX + MIN	0.55 (0.10–2.95)	0.483	0.44 (0.17–0.94)	0.022
LVX + SXT	0.27 (0.07–1.14)	0.076	0.19 (0.08–0.76)	0.035
SXT + MIN	1.14 (0.82–3.53)	0.843	0.83 (0.37–1.85)	0.646
Hospital-acquired infection	1.56 (0.32–3.99)	0.246	0.93 (0.59–1.48)	0.770
SOFA score	1.54 (1.29–1.84)	0.011	1.89 (1.25–2.12)	0.033
Carbapenem use last 90 days	3.08 (1.13–8.41)	0.028	2.02 (1.54–2.24)	0.014
Empyema	2.36 (1.08–5.15)	0.031	2.77 (2.18–3.96)	0.011

Independent variables with a *P* value <0.2 were included in the regression.

IPTW, inverse probability of treatment weighting; aOR, adjusted odds ratio; LVX, levofloxacin; SXT, trimethoprim/sulfamethoxazole; MIN, minocycline.

^a^Propensity score covariates included age, sex, BMI, hospital-acquired infection, immunocompromised status, SOFA score, year of definitive therapy initiation and the number of days from definitive antimicrobial therapy initiation to the first source control attempt.

## Discussion

This retrospective study of 190 patients with deep-seated, monomicrobial *S. maltophilia* infections evaluated outcomes between definitive monotherapy and combination therapy. Although unadjusted analyses did not demonstrate a statistically significant difference in clinical failure between treatment groups, propensity-weighted regression revealed that specific levofloxacin-based combinations, particularly levofloxacin plus minocycline and levofloxacin plus trimethoprim/sulfamethoxazole, were associated with reduced odds of clinical failure. In contrast, higher SOFA scores, carbapenem use in the preceding 90 days and the presence of empyema were independently associated with increased odds of clinical failure.

*S. maltophilia* is frequently isolated from respiratory specimens and non-sterile sites such as superficial wounds, where it may represent colonization rather than true infection.^2,4,6^ It also often appears in polymicrobial cultures alongside other virulent organisms.^4,9,10^ These factors complicate the interpretation of prior studies, particularly in pneumonia, where combination therapy has not consistently shown a benefit over monotherapy. However, such studies are limited by the potential misclassification of colonization as infection.^13,16,17,19^ To reduce this risk, we focused on monomicrobial, deep-seated infections confirmed by sterile site cultures or by blood cultures with a clinically suspected deep-seated source. We excluded cases of pneumonia and skin or soft tissue infections and required initiation of *in vitro* active therapy within 72 h of culture collection. This targeted approach was intended to minimize inclusion of colonized cases and enhance the internal validity and clinical relevance of our findings.

The 30-day all-cause mortality of 14.7% observed in our study was lower than previously reported rates for *S. maltophilia* infections, which range from 21% to 69% in critically ill patients.^7,8^ This difference may be attributable to the exclusion of pneumonia cases from our cohort, as *S. maltophilia* pneumonia has been associated with mortality rates as high as 53%.^9^ Despite the lower observed mortality, our patient population remained critically ill, as evidenced by ICU-level care in more than half of patients and median APACHE II and SOFA scores, and carried a high baseline mortality risk reflected by a Charlson Comorbidity Index of 4. The observed association between higher SOFA scores and clinical failure aligns with prior literature identifying acute physiological derangement as a strong predictor of poor outcomes.^8,24,25^

Immunocompromised patients comprised ∼23% of our population. These individuals are known to be at increased risk for S. maltophilia infection and mortality.^24,26–29^ This high-risk population has a disproportionate amount of carbapenem exposure compared to other populations, and recent carbapenem use may cause breakthrough S. maltophilia infections.^27,28^ Our study found that prior exposure to carbapenems was significantly associated with clinical failure. This finding aligns with the hypothesis that broad-spectrum antibiotics, particularly carbapenems, disrupt normal flora and promote overgrowth of intrinsically resistant organisms *such as S. maltophilia*.^27,28^ This emphasizes the importance of antimicrobial stewardship, especially in immunocompromised or previously hospitalized patients.

Patients receiving levofloxacin plus trimethoprim/sulfamethoxazole had disproportionally higher rates of clinical failure, largely driven by higher rates of 30-day mortality compared to other regimens in the unadjusted cohort. While this could raise concern about the effectiveness of the combination, these unadjusted outcomes may reflect selection bias, as more aggressive therapy is often chosen for patients with more severe illness. After adjustment for confounding variables using IPTW, levofloxacin plus minocycline and levofloxacin plus trimethoprim/sulfamethoxazole were associated with reduced odds of clinical failure. Prior studies in high-risk groups, including those with hematologic malignancies, have reported unfavourable outcomes with levofloxacin plus trimethoprim/sulfamethoxazole and raised concerns about potential antagonism, possibly due to a shared efflux pump resistance mechanism.^27,29–31^ In contrast, our adjusted outcomes indicate that this combination was not associated with harm and may be effective when baseline differences in illness severity are considered.

Although fluoroquinolone resistance has been described in S. maltophilia, particularly with prolonged therapy, our study did not evaluate serial minimum inhibitory concentrations and therefore cannot assess for treatment-emergent resistance.^27,29–31^ However, all included patients received in vitro active therapy, and the median treatment durations were similar across groups. Given the overlapping resistance mechanism between levofloxacin and trimethoprim/sulfamethoxazole, concerns about cross-resistance remain plausible.^27,29–31^ Still, our findings suggest that the combination can be considered a reasonable option when guided by susceptibility data and clinical context.

This study reinforces the importance of individualized antimicrobial selection for definitive *S. maltophilia* therapy based on *in vitro* susceptibility, infection site and patient-specific factors. Although we did not observe a statistically significant difference in clinical failure between monotherapy and combination therapy in unadjusted analyses, adjusted modelling identified reduced odds of clinical failure with specific levofloxacin-based combinations, namely levofloxacin plus minocycline and levofloxacin plus trimethoprim/sulfamethoxazole. These findings do not support broad equivalence between monotherapy and combination therapy. Given the potential for inducible fluoroquinolone resistance described in the literature, antimicrobial stewardship should prioritize thoughtful use of fluoroquinolones, particularly in patients with prolonged hospitalizations or prior fluoroquinolone exposure.^27,31^ While trimethoprim/sulfamethoxazole, levofloxacin and minocycline have historically been considered first-line agents, our findings support their continued role, especially in the combination regimens associated with improved outcomes. Recent IDSA guidance prioritizes cefiderocol and ceftazidime/avibactam plus aztreonam for *S. maltophilia*, but these agents were not utilized as definitive therapy in our cohort, limiting direct comparison with current recommendations.^14^

This study has notable strengths. It includes a relatively large cohort of patients with deep-seated, monomicrobial *S. maltophilia* infections and applies a rigorous definition of definitive therapy, limited to regimens with *in vitro* activity used for ≥70% of the total treatment duration. Inclusion criteria ensured that all patients received *in vitro* active therapy within 72 h of culture collection, with susceptibility based on CLSI breakpoints and recommended antimicrobial dosages. Confounding was addressed using IPTW-adjusted regression modelling and a time variant analysis was performed to control for evolving recommendations for management of *S. maltophilia* during the study period. In addition, by focusing on deep-seated sites and excluding polymicrobial infections, the risk of misclassification due to colonization was minimized.

Limitations should be acknowledged. This was a retrospective, single-centre study, which may limit the external validity of our findings. Although IPTW was used to control for measured confounders, residual confounding from unmeasured variables cannot be excluded. Changes to CLSI breakpoints during the study period may also introduce interpretive challenges. Specifically, the breakpoint for minocycline was lowered from ≤4 µg/mL (based on 100 mg twice daily) to ≤1 µg/mL (based on 200 mg twice daily).^22^ Susceptibility was assessed according to the breakpoint in effect at the time of culture collection, and while we limited inclusion to patients who received minocycline 200 mg twice daily, we cannot confirm that earlier isolates would be classified as susceptible under current criteria. The relatively small proportion of immunocompromised patients (22.6%) limits the generalizability of our findings to this high-risk group. Additionally, outcomes beyond 90 days were not assessed, which may be relevant for infections requiring prolonged therapy.

Prospective, randomized trials comparing monotherapy and combination therapy are needed to better define optimal treatment strategies for deep-seated *S. maltophilia* infections. Further research should investigate treatment-emergent resistance, particularly with levofloxacin during prolonged therapy, and evaluate the role of biofilm disruption strategies in improving clinical outcomes. Studies evaluating the PK/PD of trimethoprim/sulfamethoxazole and levofloxacin in combination may also clarify the utility of dual therapy in select clinical scenarios. In addition, studies assessing the effectiveness of cefiderocol and ceftazidime-avibactam plus aztreonam as definitive therapies for deep-seated *S. maltophilia* infections are warranted given their prioritization in recent treatment guidance.

### Conclusion

This study found no significant difference in clinical failure between definitive monotherapy and combination therapy in unadjusted analyses of deep-seated *S. maltophilia* infections when *in vitro* active treatment was initiated within 72 h of culture collection. However, adjusted models demonstrated that levofloxacin plus minocycline and levofloxacin plus trimethoprim/sulfamethoxazole were associated with reduced odds of clinical failure, suggesting a potential benefit of these combinations in select patients. Clinical failure was more likely among patients with higher SOFA scores, recent carbapenem exposure and the presence of empyema. Until prospective trials are available, treatment decisions should be guided by patient-specific factors, local resistance patterns and timely susceptibility data, with antimicrobial stewardship remaining central to optimal therapy.
